# Out-of-pocket expenditures on child health during an economic recession in Brazil

**DOI:** 10.11606/s1518-8787.2025059006388

**Published:** 2026-01-09

**Authors:** Marcelo Torres da Silva, Cesar Augusto Oviedo Tejada, Anderson Moreira Aristides, Alicia Matijasevich, Iná S. Santos, Andréa Dâmaso Bertoldi

**Affiliations:** I Universidade Federal de Pelotas. Programa de Pós-Graduação em Epidemiologia. Pelotas, RS, Brasil; II Universidade Federal de Pelotas. Programa de Pós-Graduação em Organizações e Mercados. Pelotas, RS, Brasil; III Universidade Federal de Alagoas. Programa de Pós-graduação em Economia. Maceió, AL, Brasil; IV Universidade de São Paulo. Departamento de Medicina Preventiva. São Paulo, SP, Brasil

**Keywords:** Health Expenditures, Children, Economic Recession, Health Economics

## Abstract

**OBJECTIVE:**

To investigate the association between the 2014–2016 economic recession in Brazil and households’ direct expenditures on child health across the distribution of such expenditures.

**METHODS:**

Using longitudinal datasets that followed children born in Pelotas, in the state of Rio Grande do Sul, in 2004 and 2015, estimates were obtained using a Double–Hurdle model, which provides one outcome for the probability of incurring any expenditure and another for the expected value of those expenses when they occur.

**RESULTS:**

The findings showed that the economic recession during this period reduced the likelihood of incurring any health-related expenditure, excluding health insurance, by 34% among 12-month-old children and by 29% among 24-month-old children. Regarding health insurance expenditures, the recession reduced the likelihood of occurrence by 37% among 12-month-olds and by a substantial 68% among 24-month-olds. As for the amount spent, given that some expenditure occurred, the economic recession was associated with an expected 15% higher total spending excluding health insurance, and a 9% lower expected spending on health insurance for 12-month-old children.

**CONCLUSIONS:**

Thus, total health expenditures, excluding health insurance, were less likely to occur during the economic recession; however, when such expenditures occurred, their expected value was higher. In contrast, spending on health insurance was lower in both analyses, reinforcing its preventive nature.

## INTRODUCTION

The analysis of out-of-pocket health expenditures usually examines the relationship between this type of spending and individual or environmental characteristics of those who make such expenditures or benefit from them.

Out-of-pocket health expenditure refers to any payment made directly by the household to obtain goods or services related to health. These payments typically include consultations with healthcare professionals, diagnostic exams, purchase of medicines (including self-prescribed ones), hospitalization bills, health insurance, and expenses related to alternative health care^1^.

Expenditures on medicines and health insurance are usually the most significant components of total health spending^4^. Regarding the determinants of these expenditures, factors such as income, educational level of the head of the household, and health status tend to strongly influence both the probability of incurring such expenses and the actual amount spent^5,6^.

Generally, studies indicate a positive relationship between individual or household income and health expenditure. However, the impact on household budget tends to be smaller at higher income levels, presenting a regressive pattern^5^. Given this association between the economic and financial situation of individuals or households and their health spending, it becomes relevant to analyze the possible impact of an economic recession on these expenditures.

The literature on the impact of economic recessions on health expenditures, although extensive, still presents gaps—especially regarding out-of-pocket spending on children’s health. Most studies focus on aggregate indicators, such as public health expenditures or private health insurance expenditures, or, when addressing out-of-pocket health spending, they tend to examine only adults^3,5,6^.

Concerning health itself, studies conducted with adults have shown that the effects of economic shocks can lead to outcomes in both directions. In some cases, improvements were observed due to the decrease in harmful behaviors resulting from financial constraints, while in others, a deterioration in health occurred, driven by stress related to job loss and difficulties in reentering the labor market^8^.

Regarding health expenditures—still focusing on adults, given the lack of studies specifically addressing children—periods of economic recession tend to particularly affect individuals from lower social classes or those without health insurance. This pattern corroborates the strong sensitivity of these expenditures to household income, as lower-income families are compelled to reduce such spending more abruptly compared to wealthier households^9^.

Economic recessions can be understood as periods of short-term declines in macroeconomic indicators, even when these indicators show relevant improvements when analyzed over the long term.

According to the *Comitê de Datação do Ciclo Econômico da Fundação Getúlio Vargas* (Brazilian Business Cycle Dating Committee of the Fundação Getúlio Vargas)^10^, Brazil experienced two major phases of the economic cycle throughout the 21^st^ century. During the first major expansion, from the third quarter of 2003 to the third quarter of 2008, the Gross Domestic Product grew for 21 consecutive quarters, accumulating a 30.2% increase. This positive phase, despite a brief interruption in 2009, resumed and sustained strong growth until mid-2014^11^.

Conversely, the second phase, marked by economic recession, occurred from the third quarter of 2014 to the fourth quarter of 2016, when the Gross Domestic Product contracted for 11 consecutive quarters, accumulating an 8.1% decline. According to many economists, this period can be considered the most severe economic recession in Brazil’s history^12^.

Thus, in an effort to understand the potential impact of an economic recession on household spending regarding child health, this study analyzed the association between the 2014–2016 economic recession in Brazil and household’s out-of-pocket health expenditures for children aged 12 and 24 months. The analysis was based on data from the Pelotas Birth Cohorts of 2004 (economic expansion phase) and 2015 (economic recession phase).

## METHODS

### Sample

This study uses data from the 2004 and 2015 Birth Cohorts of Pelotas, in the state of Rio Grande do Sul, Brazil, both conducted by the Center for Epidemiological Research at the Universidade Federal de Pelotas. These cohorts followed all live births of mothers residing in urban areas of Pelotas (including the Jardim América neighborhood, part of the adjacent municipality of Capão do Leão). The children were followed-up at several stages during childhood: perinatal, three months, 12 months, 24 months, four years, and six to seven years.

In 2004, a total of 4,263 children were eligible for the 2004 Pelotas Birth Cohort, and the mothers of 4,231 agreed to participate in the study. Follow-up rates were 96% at three months, 94% at 12 months, and 93% at 24 months^13^. In 2015, 4,387 children were born in Pelotas, of whom 4,333 were eligible and invited to participate in the 2015 Pelotas Birth Cohort. Follow-up rates were 97% at three months, 95% at 12 months, and 95% at 24 months^14^.

This study used the 12- and 24-month follow-ups from both cohorts, as these periods align with the timeframe intended for analyzing the impact of the economic context and provide complete and relevant information to such analysis. Thus, although the data originate from longitudinal studies, this analysis adopts a cross-sectional perspective across four periods (12 and 24 months of the 2004 and 2015 cohorts).

After applying necessary adjustments and excluding observations with missing or zero income, the final sample included 3,770 children from the 2004 cohort and 3,881 from the 2015 cohort at the 12-month follow-up, and 3,595 children from the 2004 cohort and 3,221 from the 2015 cohort at the 24-month follow-up.

### Variables

Five categories of child health expenditures were collected in the sample and used as dependent variables, namely: spending on medicines, medical consultations, complementary exams, other health-related items (not included in the previous categories), and health insurance (monthly premiums). These variables were obtained via questionnaires, in which participants reported the amount spent in each category over the previous 30 days. Due to the specific characteristics of health insurance expenditures for children—both in terms of the decision to spend, as such costs can be considered a form of insurance rather than an immediate need, and because they are often part of a family or business plan—analyses of this expenditure category were conducted separately^15^.

All monetary data used in the analyses were properly adjusted and updated to December 2017, which corresponds to the final follow-up date (24 months) of the 2015 cohort. Values were expressed in logarithmic form to adjust for distribution skewness.

In the analysis, the main explanatory variable is the economic recession, which is represented by a binary variable that takes the value of one for the survey years 2015–2016 and zero for the survey years 2004–2005. According to the data presented, the economic recession officially began in the second quarter of 2014 and ended in the fourth quarter of 2016. Hence, we compared household’s out-of-pocket health expenditures for children in 2015–2016 (during the economic recession) with those in 2004–2005 (during the economic expansion), considering children of the same ages and from birth cohorts in the same city.

Since the period of 2004–2016 comprises distinct phases of the economic cycle, these data do not allow the isolation of specific effects of the post-2014 economic recession. However, they do allow for the assessment of whether the recession was able to change the pattern of household’s out-of-pocket expenditures on child health that had been observed in 2004–2005, a period of strong economic expansion.

Regarding the other independent variables, those found in the predominant literature on the subject and recognized as determinants of health expenditures were included as controls^5,6^, both child- and household-related, which were available in the selected databases. These variables are as follows: total household income, stratified into quintiles for mean distribution analysis (in ascending order, with 1 being the poorest quintile and 5 the richest quintile) and expressed in logarithmic form in the estimates to adjust for theoretical distributional assumptions^16^; child’s sex (male or female); type of delivery (vaginal or cesarean); hospitalization of the child since the last follow-up (yes or no); child’s weight at birth; child’s health status as reported by the mother (categorized as healthy for responses “good,” “very good,” and “excellent”); mother’s self-reported health status (same categorization); mother’s age (in completed years); mother’s schooling (in completed years, measured at the child’s birth); mother’s self-reported race/skin color (White or Black/other); identification of the head of the household (mother, father, or other); and maternal smoking during pregnancy (yes or no).

### Analyses

After presenting the sample characteristics, and for the purpose of statistical analysis, the datasets were grouped by follow-up period, resulting in one set of observations for 12-month-old children and another for 24-month-old children. Each dataset combined observations from the 2004 and 2015 cohorts, thereby enabling an analysis of the impact of the economic context—especially the 2014–2016 economic recession—on household’s direct expenditures for child health.

Statistical analyses were performed using the Double–Hurdle Regression model^17^, which performs estimates in two stages. The first stage, using a censored sample, examines the relationship between the selected independent variables and the likelihood of incurring child health expenditures. The second stage, using a truncated sample, estimates the relationship between these variables and the expected amount of expenditure.

The term “censored” refers to the dependent variable being left-censored at zero, as a substantial number of observations take this value. Conversely, the term “truncated” refers to the subsample that includes only those who reported some type of expenditure. This distinction is necessary due to the particular nature of such spending, since many households do not incur any health-related expenditures of this type^18,19^.

The model enables the use of two sets of estimators, one for each stage of the regression. According to the prevailing theoretical framework, the first stage—which seeks to identify factors influencing the likelihood of spending—is primarily driven by non-economic factors, thus justifying the exclusion of income variables at this stage^20^.

Following this approach, the presentation of results is also divided into two parts: the first concerns the probability of spending on child health, expressed in odds ratios; the second examines the individual effect of each variable, controlling for the others, on the expected value of these expenditures when they occur, expressed in terms of marginal effects.

## RESULTS

In the 12- and 24-month follow-ups of the 2004 and 2015 cohorts, a consistent pattern can be observed in the characteristics of children and households (Table 1), with similar prevalence across most variables.

**Table 1:** Characteristics of children (and their households) participating in the 2004 and 2015 Birth Cohorts in Pelotas, state of Rio Grande do Sul (12- and 24-month follow-ups).

Variables	2004 Cohort		2015 Cohort	
	12 months (n = 3,770)	24 months (n = 3,595)	12 months (n = 3,881)	24 months (n = 3,221)
	n (%)	n (%)	n (%)	n (%)
Sex				
Female	1,810 (48.0)	1,722 (47.9)	1,906 (49.1)	1,575 (48.9)
Male	1,960 (52.0)	1,873 (52.1)	1,975 (50.9)	1,646 (51.1)
Type of delivery				
Vaginal	2,062 (54.7)	1,981 (55.1)	1,354 (34.9)	1,092 (33.9)
Cesarean section	1,708 (45.3)	1,614 (44.9)	2,527 (65.1)	2,129 (66.1)
Mother’s race/skin color				
White	2,760 (73.2)	2,628 (73.1)	2,759 (71.1)	2,338 (72.6)
Non-white	1,010 (26.8)	967 (26.9)	1,122 (28.9)	883 (27.4)
Smoking during pregnancy				
Yes	1,014 (26.9)	974 (27.1)	617 (15.9)	473 (14.7)
No	2,756 (73.1)	2,621 (72.9)	3,264 (84.1)	2,748 (85.3)
Head of the family				
Father	2,613 (69.3)	2,423 (67.4)	2,169 (55.9)	2,029 (63.0)
Mother	467 (12.4)	532 (14.8)	1,153 (29.7)	944 (29.3)
Other	690 (18.3)	640 (17.8)	559 (14.4)	248 (7.7)
Child’s health				
Excellent	1,512 (40.1)	1,176 (32.7)	1,859 (47.9)	1,334 (41.4)
Very good	818 (21.7)	830 (23.1)	900 (23.2)	892 (27.7)
Good	1,191 (31.6)	1,291 (35.9)	935 (24.1)	776 (24.1)
Regular	230 (6.1)	284 (7.9)	175 (4.5)	203 (6.3)
Poor	19 (0.5)	14 (0.4)	12 (0.3)	16 (0.5)
Mother’s health				
Excellent	792 (21.0)	662 (18.4)	819 (21.1)	583 (18.1)
Very good	599 (15.9)	618 (17.2)	827 (21.3)	696 (21.6)
Good	1,761 (46.7)	1,718 (47.8)	1,715 (44.2)	1,350 (41.9)
Regular	566 (15.0)	543 (15.1)	474 (12.2)	522 (16.2)
Poor	52 (1.4)	54 (1.5)	46 (1.2)	70 (2.2)

However, it is worth noting the significant drop in the prevalence of vaginal deliveries in the 2015 cohort—34.9% and 33.9% at the 12- and 24-month follow-ups, respectively—compared to 54.7% and 55.1% in the 2004 cohort. A similar downward trend was also observed in smoking during pregnancy, with a significant decrease in its prevalence in the 2015 cohort.

The percentage of families reporting any type of health expenditure was 52.9% for 12-month-old children and 49.0% for 24-month-old children in the 2004 cohort, and 49.8% and 47.4%, respectively, in the 2015 cohort. Expenditures on medicines were the most frequent among the categories of health spending analyzed. As shown in Table 2, in the 2004 cohort, this type of expenditure had a prevalence of 49.0% among 12-month-old children and 47.0% among 24-month-old children, whereas in the 2015 cohort, the corresponding figures were 43.5% and 44.3%, respectively.

**Table 2:** Average monthly expenditure in Brazilian reais, percentage of households reporting expenditures, and average conditional monthly expenditurea in reais for the five categories of child health-related expenses in the 2004 and 2015 Birth Cohorts of Pelotas, state of Rio Grande do Sul (12- and 24-month follow-ups).

Type of expense (Follow-up)	Cohort	Average monthly expenditure (R$)	Percentage of households reporting expenditures	Average conditional monthly expenditure (R$)
Expenses with medicines				
12 months				
	2004 (n = 3,770)	32.72	49.0%	64.42
	2015 (n = 3,881)	34.19	43.5%	76.40
24 months				
	2004 (n = 3,477)	31.10	47.0%	64.19
	2015 (n = 3,196)	34.47	44.3%	74.76
Expenses with medical consultations				
12 months				
	2004 (n = 3,770)	7.99	13.5%	58.30
	2015 (n = 3,881)	22.77	16.8%	124.29
24 months				
	2004 (n = 3,477)	6.19	9.3%	64.39
	2015 (n = 3,196)	19.43	11.5%	146.91
Expenses with complementary exams				
12 months				
	2004 (n = 3,770)	2.26	4.6%	46.31
	2015 (n = 3,881)	5.77	5.5%	105.26
24 months				
	2004 (n = 3,477)	1.37	2.7%	44.01
	2015 (n = 3,196)	6.78	4.5%	142.17
Expenses with health insurance				
12 months				
	2004 (n = 3,476)	30.91	27.9%	80.09
	2015 (n = 3,261)	40.42	32.2%	106.93
24 months				
	2004 (n = 3,137)	29.61	28.8%	77.65
	2015 (n = 3,131)	36.45	24.5%	120.33
Other health-related expenses^b^				
12 months				
	2004 (n = 3,770)	0.57	0.4%	147.86
	2015 (n = 3,881)	2.31	0.8%	147.36
24 months				
	2004 (n = 3,477)	0.65	0.3%	121.46
	2015 (n = 3,196)	5.75	1.2%	308.66

^a^ Average monthly expenditure among households that reported any type of expenditure.

^b^ Other health-related expenses, excluding dental expenses.

However, this pattern was not maintained for other types of expenditures. In the 2015 cohort, a higher percentage of families reported spending on medical consultations, complementary tests, and other health-related items for their children compared to the 2004 cohort.

As for health insurance expenditures, the results showed variations. Among 12-month-old children, the occurrence was higher in the 2015 cohort (32.2%) compared to the 2004 cohort (27.9%). However, for 24-month-old children, the opposite pattern was observed: 28.8% of families in the 2004 cohort reported such expenses, compared with 24.5% in the 2015 cohort.

Regarding the amount spent, expenditures were higher in the 2015 cohort across nearly all categories of child health expenses, for both 12- and 24-month-old children. These values were significantly higher for medical consultations and complementary tests (Table 2).

Regarding conditional spending, which was analyzed only among families who incurred any health-related costs, there were also differences between the 2004 and 2015 cohorts, with higher values in the latter. While spending on medicines was only slightly higher, expenditures on medical consultations more than doubled for both age groups, and spending on complementary exams for 24-month-old children increased by an impressive 223%. For health insurance expenses among 24-month-olds, the 2015 cohort showed approximately 55% higher spending than the one on the 2004 cohort.

Another aspect analyzed was the distribution of average expenditures on child health by household income quintile. As shown in Figure, there is a clear dispersion of total spending on child health in the highest income quintile compared to the others, especially in the 2015 cohort. This pattern reflects higher expenditures by the top income quintile on health insurance and medical consultations for their children. Across all scenarios analyzed, the average total spending of the highest income quintile was more than twice that of the fourth quintile and five times that of the first quintile.

**Figure:**
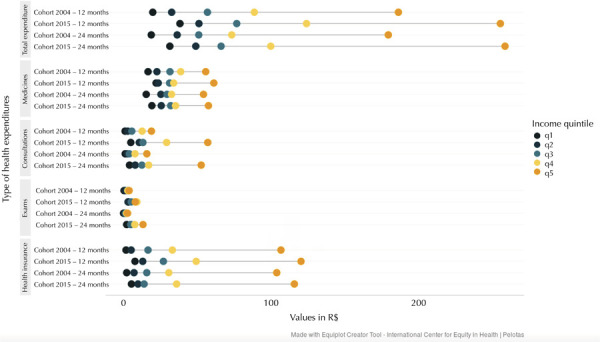
Average monthly health expenditures for children in the 2004 and 2015 Birth Cohorts of Pelotas, state of Rio Grande do Sul, by type of expenditure and income quintiles.

Regarding estimates from the first stage, which analyzes the probability of incurring any expenditure, the unfavorable economic scenario (2015) presented a 34% lower likelihood of total health spending (excluding health insurance) for 12-month-old children and a 29% lower likelihood for 24-month-old children (Table 3). For child health insurance expenditures, the economic shock had a more pronounced impact, with a 37% lower likelihood for 12-month-olds and a 68% lower likelihood for 24-month-olds. Among control variables, cesarean delivery and maternal race/skin color were positively associated with expenditures in all analyses, whereas the child being healthy was inversely related to total spending, excluding health insurance.

**Table 3:** Odds ratio (95%CI) of the economic scenario on the probability of child health-related expenditures in the 12- and 24-month follow-ups of the 2004 and 2015 Birth Cohorts in Pelotas, state of Rio Grande do Sul.

	12-month follow-up		24-month follow-up	
	Total expenditure^a^ (n = 7,651)	Health insurance expenses (n = 6,737)	Total expenditure^a^ (n = 6,918)	Health insurance expenses (n = 6,546)
Economic scenario				
2004	-	-	-	-
2015^b^	0.66* (0.60 to 0.73)	0.63* (0.55 to 0.72)	0.71* (0.64 to 0.79)	0.32* (0.28 to 0.37)

95%CI: 95% confidence interval.

Note: controlled for child’s sex, type of delivery, child’s birth weight, hospitalization, child considered healthy, number of residents in the household, mother considered healthy, head of the family, mother’s age, mother’s schooling, mother’s race/skin-color, and smoking during pregnancy.

^a^ Except for health insurance expenses.

^b^ Negative scenario, economic shock.

* p-value < 0.001.

In the second stage of estimation, which considers the expected value of child health expenditures conditional on having incurred any spending, the economic recession had a significant impact. For 12-month-old children, total health spending, excluding health insurance, was 15% higher during the recession, whereas for 24-month-olds it was 14% higher. However, for health insurance expenditures, the expected value decreased by 9% for 12-month-old children during the recession (Table 4).

**Table 4:** Marginal effect (95%CI) of the economic scenario on the expected value of child health-related expenditures in the 12- and 24-month follow-ups of the 2004 and 2015 Birth Cohorts in Pelotas, state of Rio Grande do Sul.

-	12-month follow-up		24-month follow-up	
	Total expenditure^a^ (n = 3,925)	Health insurance expenses (n = 2,020)	Total expenditure^a^ (n = 3,314)	Health insurance expenses (n = 1,744)
Economic scenario				
2004	-	-	-	-
2015^b^	0.15* (0.07 to 0.22)	-0.09** (-0.17 to -0.01)	0.14** (0.06 to 0.22)	-0.01 (-0.10 to 0.08)

95%CI: 95% confidence interval.

Note: controlled for household income, child’s sex, type of delivery, child’s birth weight, hospitalization, child considered healthy, mother considered healthy, head of the family, mother’s age, mother’s schooling, mother’s race/skin color, and smoking during pregnancy.

^a^ Except for health insurance expenses.

^b^ Negative scenario, economic shock.

* p-value < 0.001.

** p-value < 0.05.

Finally, regarding control variables, household income and cesarean delivery were positively associated with the expected value of expenditures in all scenarios: higher household income or cesarean delivery increased the expected expenditure. The child being healthy was inversely related to total spending, excluding health insurance, reducing the expected expenditure as the child’s health status improved.

## DISCUSSION

The discussion of the results found requires some adaptation, since, to the best of our knowledge, no study to date has examined household’s out-of-pocket expenditures on child health during an economic recession—only adult health expenditures have been analyzed^21^. Thus, these results will be used for comparison purposes, while clearly noting the important distinction.

Our study found a lower probability of households incurring out-of-pocket health expenditures for children during an economic recession; however, when spending did occur, the expected amount was higher.

The reduced likelihood of incurring any health expenditure during a recession aligns with findings from other studies. Nevertheless, in those studies, the expected value of spending was also lower during the economic crisis^21^, unlike what was observed in our analysis.

This divergent result, showing an increasing trend in the expected value of expenditures when incurred during the crisis, may reflect either a deterioration of the public health system during these periods due to reduced public investment^26^ or a general increase in the prices of health goods and services.

A possible explanation for this result is the implementation of fiscal austerity measures by the government during the recession, including cuts to municipal health expenditures. Consequently, as the economic crisis worsens the health condition of the population and reduced the quantity and quality of available health goods and services, households may respond by increasing their out-of-pocket health expenditures—a pattern also observed in Greece during the 2008 financial crisis^24^.

It is important to highlight that this pattern did not apply to health insurance expenditures, as the economic crisis was associated with a lower probability of occurrence and a lower expected value of spending. This underscores the distinct behavior of health insurance compared to other types of health expenditures. Being a form of “insurance” with monthly payments, families can choose to cancel their coverage when their income drops, hoping that no health issues should require its use^27^.

In this context, a lower probability of health insurance spending for children during the economic crisis—37% in 12-month-olds and a striking 68% in 24-month-olds—supports the preventive nature of health plans^27^. Therefore, it is natural to observe higher prevalence in the first months of life, which require various medical consultations and exams, followed by a sharp decline as children grow older due to the economic downturn.

This study has limitations typical for research using self-reported data on out-of-pocket expenditures. Information bias regarding health insurance spending warrants particular attention, as the amount actually spent on the child is often included in the total paid for a family plan or partially subsidized by the parents’ employer.

Another important limitation of this study is that it analyzes health spending behavior in the context of universal healthcare access, in which many families may incur no expenditures or adjust their behavior freely depending on the use of this benefit. Potential differences in social, economic, and cultural conditions across locations should also be considered, requiring caution when generalizing the results to broader contexts.

It should be noted that the programs *Brasil Sorridente*, aimed at improving oral health, and *Farmácia Popular do Brasil*, designed to expand access to medicines, were both launched in 2004. These represent the most recent political initiatives with a significant impact on Brazilian families’ health-related expenditures.

Thus, since these programs affected both periods analyzed and there were no other significant changes in public policies altering the scope of the health system, we can reasonably assume that the Brazilian Unified Health System (SUS) influenced both periods equally.

Finally, our finding of an opposite trend in the expected value of private health expenditures during economic crises, compared to previous studies focused on the topic, while explainable, highlights the need for further research to better understand this relationship and the impact of public health expenditures.

Future studies could also examine more specifically the role of health insurance spending for children and further investigate total health expenditures, aiming to explore direct interactions between the economic crisis and its determinants, which in our analysis were used only as control variables.

## Data Availability

The questionnaires and interview guides for all follow-up visits are available from [http://www.epidemio-ufpel.org.br/coorte-2004] and [http://www.epidemio-ufpel.org.br/coorte-2015].
