# Supplementary material for: Out-of-pocket expenditures on child health during an economic recession in Brazil
Source: Rev Saude Publica. 2026 Jan 9;59:e48. doi: 10.11606/s1518-8787.2025059006388 (PMC12713523; doi:10.11606/s1518-8787.2025059006388)
Supplement: Translation

# Translation: Gastos diretos das famílias com a saúde infantil durante recessão econômica no Brasil

**Authors:** Marcelo Torres da Silva, Cesar Augusto Oviedo Tejada, Anderson Moreira Aristides, Alicia Matijasevich, Iná S. Santos, Andréa Dâmaso Bertoldi

**Affiliations:** I Universidade Federal de Pelotas. Programa de Pós-Graduação em Epidemiologia. Pelotas, RS, Brasil; II Universidade Federal de Pelotas. Programa de Pós-Graduação em Organizações e Mercados. Pelotas, RS, Brasil; III Universidade Federal de Alagoas. Programa de Pós-graduação em Economia. Maceió, AL, Brasil; IV Universidade de São Paulo. Departamento de Medicina Preventiva. São Paulo, SP, Brasil

**Keywords:** Gastos com Saúde, Crianças, Recessão Econômica, Economia da Saúde

## Abstract

**OBJETIVO:**

Investigar a associação entre a recessão econômica no Brasil de 2014 a 2016 e os gastos diretos das famílias com a saúde das crianças, ao longo da distribuição desses gastos.

**MÉTODOS:**

Utilizando bases de dados longitudinais que acompanharam crianças nascidas em Pelotas (RS) nos anos de 2004 e 2015, foram realizadas estimativas por meio de um modelo *Double-Hurdle*, o qual fornece um resultado sobre a probabilidade de ser realizado gasto e outro sobre o valor esperado desses gastos quando ocorrerem.

**RESULTADOS:**

Os resultados apontaram que a recessão econômica durante esse período reduziu em 34% a chance de ocorrência de gasto com saúde, exceto plano de saúde, para crianças de 12 meses, enquanto diminui essa chance em 29% para as crianças de 24 meses. Em relação aos gastos com plano de saúde, a recessão econômica reduziu em 37% a chance de ocorrência entre crianças de 12 meses, e em expressivos 68% entre crianças de 24 meses. Quanto ao montante do gasto, dado que algum dispêndio fora realizado, a recessão econômica está associada a um valor esperado 15% maior nos gastos totais exceto plano de saúde, e 9% menor nos gastos com plano de saúde para crianças de 12 meses.

**CONCLUSÕES:**

Deste modo, o gasto total em saúde, exceto com plano de saúde, apresentou probabilidade menor de ocorrência durante período de recessão econômica, contudo, com valor esperado superior quando realizado. Por sua vez, o gasto com plano de saúde se mostrou menor nas duas análises, corroborando seu caráter preventivo.

## INTRODUÇÃO

A análise dos gastos diretos em saúde costuma examinar a relação deste tipo de dispêndio com características individuais e ambientais de quem realiza ou daquele que vem a se beneficiar de tal gasto.

O gasto direto em saúde se refere a todo pagamento feito diretamente pela família visando obter algum bem ou serviço ligado à saúde. Estes pagamentos geralmente incluem consultas com profissionais de saúde, exames diagnósticos, compra de medicamentos (incluindo autoprescritos), contas de hospitalizações, planos de saúde e gastos com cuidados alternativos de saúde^1^.

Os gastos com medicamentos e com plano de saúde costumam ser aqueles mais expressivos entre os gastos com saúde^4^. Em relação aos determinantes desses gastos, fatores como renda, nível educacional do chefe da família e estado de saúde costumam mostrar uma forte influência tanto na probabilidade de realização quanto no montante do gasto efetivamente realizado^5,6^.

Em termos gerais, os estudos indicam uma relação positiva entre a renda do indivíduo, ou do domicílio, e o gasto em saúde, contudo, o impacto no orçamento familiar tende a ser menor nos níveis de renda superiores, apresentando um comportamento regressivo^5^. Dada essa associação entre a situação econômico-financeira do indivíduo ou do domicílio e o gasto com saúde, torna-se relevante a análise de um possível impacto nestes gastos ocasionado por um período de recessão econômica.

A literatura sobre o impacto da recessão econômica nos gastos com saúde, apesar de extensa, deixa lacunas, especialmente no que se refere aos gastos diretos das famílias com a saúde das crianças, visto que grande maioria dos trabalhos se concentram em indicadores agregados, como gastos públicos em saúde ou despesas de planos de saúde, ou ainda, quando tratando-se de gastos diretos em saúde, apenas em relação aos adultos^3,5,6^.

Quanto à saúde propriamente dita, os estudos realizados com adultos mostraram que os efeitos de choques econômicos geram resultados em ambas as direções. Em certos casos foi observada melhora baseada na diminuição de comportamentos nocivos por incapacidade financeira, enquanto em outros, uma deterioração da saúde causada por estresse com perda de emprego e dificuldade de recolocação no mercado de trabalho^8^.

Quanto ao gasto com saúde, ainda no que concerne aos adultos, em virtude da falta de trabalhos específicos para crianças, os períodos de recessão econômica costumam afetar especialmente aqueles das classes sociais mais baixas ou que não possuem plano de saúde. Este comportamento corrobora a forte sensibilidade existente na relação destes gastos com a renda familiar, visto que são compelidos a cortá-los de forma mais abrupta quando comparados às famílias mais ricas^9^.

As recessões econômicas podem ser vistas como períodos de quedas dos indicadores macroeconômicos no curto prazo, ainda que estes indicadores estejam apresentando relevantes melhorias quando analisados no longo prazo.

Segundo o Comitê de Datação do Ciclo Econômico da Fundação Getúlio Vargas^10^, no Brasil, ocorreram duas grandes fases do ciclo econômico ao longo do século XXI. Durante a primeira grande expansão econômica, do terceiro trimestre de 2003 ao terceiro trimestre de 2008, o Produto Interno Bruto cresceu por 21 trimestres seguidos, acumulando um aumento de 30,2%. Esta fase positiva, mesmo com breve interrupção em 2009, retomou e manteve o forte crescimento até meados de 2014^11^.

Por outro lado, a segunda fase, de recessão econômica, ocorreu do terceiro trimestre de 2014 ao quarto trimestre de 2016, quando houve uma contração do Produto Interno Bruto por 11 trimestres seguidos, acumulando uma queda de 8,1%. Na visão de muitos economistas, esta pode ser considerada a mais severa recessão econômica na história do Brasil^12^.

Assim, buscando entender o potencial impacto ocasionado por uma recessão econômica nos gastos das famílias na saúde das crianças, o presente estudo analisou a associação da recessão econômica brasileira dos anos 2014 a 2016, com os gastos diretos das famílias na saúde das crianças de 12 e 24 meses, utilizando dados obtidos nas Coortes de Nascimentos de Pelotas (RS) de 2004 (fase de expansão econômica) e 2015 (fase de recessão econômica).

## MÉTODOS

### Amostra

O estudo utiliza dados das Coortes de Nascimentos de Pelotas (RS), Brasil, dos anos de 2004 e 2015, ambas realizadas pelo Centro de Pesquisas Epidemiológicas da Universidade Federal de Pelotas. Estas coortes acompanharam todos os nascidos vivos de mães residentes na zona urbana da cidade de Pelotas (RS) (juntamente com o bairro Jardim América pertencente ao Município do Capão do Leão, adjacente a Pelotas), tendo sido realizados durante a infância os seguintes acompanhamentos: perinatal; 3 meses; 12 meses; 24 meses; 4 anos e 6–7 anos.

No ano de 2004, nasceram 4.263 crianças elegíveis para a Coorte de Nascimentos de Pelotas de 2004, sendo que as mães de 4.231 aceitaram participar do estudo. Nos acompanhamentos seguintes, participaram 96% das crianças aos 3 meses, 94% aos 12 meses e 93% aos 24 meses^13^. No ano de 2015, nasceram 4.387 crianças na cidade de Pelotas (RS), sendo 4.333 elegíveis e convidadas a participarem da Coorte de Nascimentos de Pelotas de 2015, permanecendo nos acompanhamentos seguintes 97% aos 3 meses, 95% aos 12 meses e 95% aos 24 meses^14^.

Foram utilizados neste estudo os acompanhamentos de 12 e 24 meses, de ambas as coortes, estando as coortes, no lapso temporal em que se pretende analisar o impacto gerado pelo cenário econômico, bem como por possuírem a completude das informações relevantes para tal análise. Deste modo, trata-se de uma análise oriunda de dados de coortes de acompanhamentos, entretanto, neste caso, sob a ótica de recortes transversais em quatro períodos (12 e 24 meses das coortes de 2004 e 2015).

Após os ajustes necessários, sendo excluídas as observações com renda nula ou não informada, restaram 3.770 crianças da Coorte de 2004 e 3.881 da Coorte de 2015, no acompanhamento de 12 meses, e 3.595 crianças da Coorte de 2004 e 3.221 da Coorte de 2015, no acompanhamento de 24 meses.

### Variáveis

Como variáveis dependentes, foram utilizados os cinco grupos de gastos com a saúde das crianças coletados na amostra utilizada, quais sejam: gastos com medicamentos, gastos com consultas médicas, gastos com exames complementares, gastos com outros itens relacionados à saúde (não enquadrados nos grupos de gastos anteriores) e a gastos com plano de saúde (mensalidade). Essas variáveis foram obtidas por meio da realização de questionários, pelos quais os participantes das coortes informaram o valor de seus gastos nestas rubricas nos últimos 30 dias. Em virtude das particularidades do gasto com plano de saúde para as crianças, tanto em termos da decisão de gastar, visto que pode ser entendido como um seguro e não como necessidade, quanto por geralmente vir agrupado em algum plano familiar ou empresarial, as análises quanto a este tipo de gasto foram feitas de forma separada^15^.

Todos os dados monetários utilizados nas análises foram devidamente corrigidos e atualizados até dezembro de 2017, que foi a data final do acompanhamento de 24 meses da Coorte de 2015, e utilizados em termos de logaritmo como forma de ajuste de distribuição.

Na análise, tem-se que a principal variável explicativa é a recessão econômica, a qual é representada por uma variável binária que toma o valor um para identificar os anos de pesquisa 2015–2016 e zero para os anos de pesquisa de 2004–2005. Conforme os dados apresentados, o período de recessão econômica começou oficialmente no segundo trimestre de 2014 e terminou no quarto trimestre de 2016, assim, comparamos os gastos diretos com saúde das crianças em 2015–2016 (durante a recessão econômica) com os gastos diretos com saúde das crianças em 2004–2005 (durante a expansão econômica) – com crianças das mesmas idades e de coortes de nascimentos da mesma cidade.

Como o período de 2004 a 2016 perfaz fases distintas do ciclo econômico, esses dados não seriam capazes de medir separadamente os impactos da recessão econômica a partir de 2014, mas permitem sim avaliar se esta foi capaz de mudar o comportamento do gasto direto das famílias com a saúde das crianças que fora observado nos anos 2004–2005, de forte expansão econômica.

Quanto às outras variáveis independentes, foram utilizadas como controles aquelas encontradas na literatura dominante sobre o tema e conhecidas como determinantes destes gastos com saúde^5,6^, tanto da criança quanto do domicílio, e que faziam parte dos bancos de dados selecionados. São estas: a renda agregada do domicilio, estratificada em quintis para análise de distribuição das médias (em ordem crescente, sendo 1 o quintil mais pobre e 5 o quintil mais rico) e na forma de logaritmo nas estimações para ajuste de distribuição por proposição teórica^16^; o sexo da criança (masculino ou feminino); o tipo de parto (normal ou cesariana); hospitalização da criança desde o último acompanhamento (sim ou não); peso da criança ao nascer; estado de saúde da criança referido pela mãe (categorizado como saudável para as respostas “bom”, “muito bom” e “excelente”); estado de saúde da mãe autorreferido (mesmo critério do anterior); idade da mãe (em anos completos); escolaridade da mãe (em anos completos e medida no momento do nascimento da criança); cor da pele da mãe autorreferida (branca e preta/outra); indicação do chefe da família (mãe, pai ou outro); tabagismo materno durante a gravidez (sim ou não).

### Análises

Após a apresentação das características da amostra, e para fins das análises estatísticas, os bancos foram agrupados por acompanhamentos, restando um conjunto de observações para as crianças de 12 meses e outro para as crianças de 24 meses, ambos com observações conjuntas das Coortes de 2004 e 2015, permitindo assim a análise do impacto do cenário econômico, especialmente da recessão econômica do período 2014 a 2016, nos gastos direto com saúde das crianças.

As análises estatísticas foram realizadas por meio do modelo *Double-Hurdle Regression* ^17^, que realiza as estimações em duas etapas, sendo que a primeira utiliza a amostra censurada, buscando identificar a relação das variáveis independentes escolhidas com a chance de ser realizado gasto com saúde das crianças, e a segunda, utilizando a amostra truncada, buscando identificar a relação das variáveis escolhidas sobre o valor esperado do gasto.

Diz-se censurada pois a variável dependente apresenta censura à esquerda, qual seja, no valor zero, com um número relevante de observações neste ponto, enquanto truncada indica a subamostra somente com aqueles que realizaram algum tipo de gasto. Tal diferenciação se faz necessária em virtude da peculiaridade destes tipos de gastos, uma vez que muitas famílias não realizam qualquer tipo de gasto dessa natureza^18,19^.

O modelo permite a utilização de dois conjuntos de estimadores para cada parte da regressão, de modo que a teoria dominante sobre o tema admite que a primeira etapa, aquela que busca identificar os fatores que influenciam a probabilidade de gastar, seria uma função de fatores não econômicos, justificando assim a ausência de variáveis de renda neste momento^20^.

Seguindo esta linha, a apresentação dos resultados também se dá em duas etapas, sendo a primeira referente a probabilidade de ser realizado o gasto com a saúde das crianças, apresentada em termos de razão de chances, e a segunda na influência individual de cada variável, controladas as demais, sobre o valor esperado desses gastos, quando realizados, apresentada em termos de efeito marginal.

## RESULTADOS

Nos acompanhamentos analisados, de 12 e 24 meses das Coortes de Nascimentos de 2004 e 2015, é possível perceber um padrão nas características das crianças e dos domicílios (Tabela 1), com prevalências semelhantes na maioria das variáveis.

**Tabela 1:** Características das crianças (e de seus domicílios) participantes nas Coortes de Nascimentos de 2004 e 2015 de Pelotas (RS) (acompanhamentos de 12 e 24 meses).

Variáveis	Coorte 2004		Coorte 2015	
	12 meses (n = 3.770)	24 meses (n = 3.595)	12 meses (n = 3.881)	24 meses (n = 3.221)
	n (%)	n (%)	n (%)	n (%)
Sexo				
Feminino	1.810 (48,0)	1.722 (47,9)	1.906 (49,1)	1.575 (48,9)
Masculino	1.960 (52,0)	1.873 (52,1)	1.975 (50,9)	1.646 (51,1)
Tipo de parto				
Normal	2.062 (54,7)	1.981 (55,1)	1.354 (34,9)	1.092 (33,9)
Cesariana	1.708 (45,3)	1.614 (44,9)	2.527 (65,1)	2.129 (66,1)
Cor da pele da mãe				
Branca	2.760 (73,2)	2.628 (73,1)	2.759 (71,1)	2.338 (72,6)
Não branca	1.010 (26,8)	967 (26,9)	1.122 (28,9)	883 (27,4)
Fumo na gravidez				
Sim	1.014 (26,9)	974 (27,1)	617 (15,9)	473 (14,7)
Não	2.756 (73,1)	2.621 (72,9)	3.264 (84,1)	2.748 (85,3)
Chefe da família				
Pai da criança	2.613 (69,3)	2.423 (67,4)	2.169 (55,9)	2.029 (63,0)
Mãe da criança	467 (12,4)	532 (14,8)	1.153 (29,7)	944 (29,3)
Outro	690 (18,3)	640 (17,8)	559 (14,4)	248 (7,7)
Saúde da criança				
Excelente	1.512 (40,1)	1.176 (32,7)	1.859 (47,9)	1.334 (41,4)
Muito boa	818 (21,7)	830 (23,1)	900 (23,2)	892 (27,7)
Boa	1.191 (31,6)	1.291 (35,9)	935 (24,1)	776 (24,1)
Regular	230 (6,1)	284 (7,9)	175 (4,5)	203 (6,3)
Ruim	19 (0,5)	14 (0,4)	12 (0,3)	16 (0,5)
Saúde da mãe da criança				
Excelente	792 (21,0)	662 (18,4)	819 (21,1)	583 (18,1)
Muito boa	599 (15,9)	618 (17,2)	827 (21,3)	696 (21,6)
Boa	1.761 (46,7)	1.718 (47,8)	1.715 (44,2)	1.350 (41,9)
Regular	566 (15,0)	543 (15,1)	474 (12,2)	522 (16,2)
Ruim	52 (1,4)	54 (1,5)	46 (1,2)	70 (2,2)

Entretanto, cabe destacar a expressiva queda na prevalência de parto normal nas crianças da Coorte de 2015, com 34,9% e 33,9% nos acompanhamentos de 12 e 24 meses, respectivamente, frente aos 54,7% e 55,1% encontrados nos acompanhamentos da Coorte de 2004. Esta divergência também pode ser percebida em relação ao fumo durante a gravidez, com queda igualmente expressiva em sua prevalência na Coorte de 2015.

O percentual destas famílias que realizou algum tipo de gasto com saúde foi de 52,9% para crianças de 12 meses e de 49,0% para crianças de 24 meses na Coorte de 2004, e de 49,8% e 47,4%, respectivamente, na Coorte de 2015. O gasto com medicamentos mostrou-se o mais frequente entre as modalidades de gastos com saúde analisadas. Como pode ser visto na Tabela 2, na Coorte de 2004, este tipo de gasto teve prevalência de 49,0% nas crianças de 12 meses e de 47,0% nas crianças de 24 meses, enquanto que, na Coorte de 2015, foi de 43,5% e 44,3% nas mesmas idades.

**Tabela 2:** Gasto mensal médio em reais, percentual de famílias que realizaram gasto e gasto mensal médio condicionadoa em reais, para as cinco modalidades de gastos relacionados à saúde das crianças nas Coortes de Nascimentos de 2004 e 2015 de Pelotas (RS) (acompanhamentos de 12 e 24 meses).

Tipo de gasto (Acompanhamento)	Coorte	Gasto mensal médio (R$)	Percentual de famílias com gasto	Gasto mensal médio condicionado (R$)
Gasto com medicamentos				
12 meses				
	2004 (n = 3.770)	32,72	49,0%	64,42
	2015 (n = 3.881)	34,19	43,5%	76,40
24 meses				
	2004 (n = 3.477)	31,10	47,0%	64,19
	2015 (n = 3.196)	34,47	44,3%	74,76
Gasto com consultas médicas				
12 meses				
	2004 (n = 3.770)	7,99	13,5%	58,30
	2015 (n = 3.881)	22,77	16,8%	124,29
24 meses				
	2004 (n = 3.477)	6,19	9,3%	64,39
	2015 (n = 3.196)	19,43	11,5%	146,91
Gasto com exames complementares				
12 meses				
	2004 (n = 3.770)	2,26	4,6%	46,31
	2015 (n = 3.881)	5,77	5,5%	105,26
24 meses				
	2004 (n = 3.477)	1,37	2,7%	44,01
	2015 (n = 3.196)	6,78	4,5%	142,17
Gasto com plano de saúde				
12 meses				
	2004 (n = 3.476)	30,91	27,9%	80,09
	2015 (n = 3.261)	40,42	32,2%	106,93
24 meses				
	2004 (n = 3.137)	29,61	28,8%	77,65
	2015 (n = 3.131)	36,45	24,5%	120,33
Outros gastos relacionados à saúde^b^				
12 meses				
	2004 (n = 3.770)	0,57	0,4%	147,86
	2015 (n = 3.881)	2,31	0,8%	147,36
24 meses				
	2004 (n = 3.477)	0,65	0,3%	121,46
	2015 (n = 3.196)	5,75	1,2%	308,66

^a^ Gasto mensal médio entre as famílias que realizaram algum tipo de gasto.

^b^ Outros gastos com saúde exceto odontológicos.

No entanto, esse padrão não se manteve para outros tipos de gastos. Na Coorte de 2015, um maior percentual de famílias gastou com consultas médicas, exames complementares e outros itens relacionados à saúde das crianças, em comparação com a Coorte de 2004.

Quanto ao gasto com plano de saúde, os resultados mostraram variações. Para as crianças de 12 meses, a ocorrência foi maior na Coorte de 2015, com 32,2% contra 27,9% para as da Coorte de 2004. No caso das crianças de 24 meses, a ocorrência foi maior entre os participantes da Coorte de 2004, com 28,8% frente a 24,5% entre os da Coorte de 2015.

Quanto ao valor do gasto, este foi superior à Coorte de 2015 em praticamente todas as modalidades de gastos com saúde das crianças – tanto de 12 meses quanto de 24 meses. Estes valores foram expressivamente superiores nos gastos com consultas médicas e com exames complementares (Tabela 2).

Em relação ao gasto condicionado, aquele analisado somente para as famílias que tiveram algum gasto com saúde, igualmente, houve diferença entre as Coortes de 2004 e 2015, sendo mais expressivo nesta última. Este valor foi levemente superior em relação ao gasto com medicamentos, contudo, apresentou acréscimo superior a 100% nos gastos com consultas médicas para ambas as idades e impactantes 223% nos gastos com exames complementares para crianças de 24 meses. Quanto ao gasto com plano de saúde para as crianças de 24 meses, a Coorte de 2015 apresentou resultado aproximadamente 55% superior ao de 2004.

Outro ponto analisado foi a distribuição da média dos gastos com saúde das crianças, separada por quintil de renda das famílias. Nesta, podemos ver na Figura a ocorrência de uma clara dispersão do gasto total em saúde das crianças do quintil superior de renda em relação aos demais, especialmente na Coorte de 2015. Tal fato se mostra como consequência de um maior gasto do quintil superior de renda nos gastos com plano de saúde e consultas médicas das crianças. A média do gasto total do último quintil de renda foi superior a 2 vezes a média do quarto quintil e a 5 vezes a média do primeiro quintil, em todos os cenários analisados.

**Figura:** Média dos gastos mensais em saúde das crianças das Coortes de Nascimentos de Pelotas (RS) de 2004 e 2015 por tipo de gasto e quintis de renda.

Quanto as estimações realizadas na primeira etapa, aquela que analisa a probabilidade de ser realizado algum gasto, o cenário econômico desfavorável (ano de 2015) apresentou uma chance 34% menor de realização de gasto total em saúde (exceto plano de saúde) para crianças de 12 meses, e 29% menor para crianças de 24 meses (Tabela 3). Já no gasto com plano de saúde das crianças, este choque econômico apresentou impacto mais significativo, indicando uma chance 37% menor de realização para crianças de 12 meses e 68% menor para as de 24 meses. Em relação aos controles utilizados, parto por cesariana e cor da pele da mãe branca, apresentaram relação direta com o gasto em todas as análises, enquanto que “a criança ser saudável” apresentou relação inversa com o gasto total, exceto plano de saúde.

**Tabela 3:** Razão de chances (IC95%) do cenário econômico na probabilidade de ser realizado gasto com saúde das crianças nos acompanhamentos de 12 e 24 meses, nas Coortes de Nascimentos de Pelotas (RS) de 2004 e 2015.

	Acompanhamento de 12 meses		Acompanhamento de 24 meses	
	Gasto total^a^ (n = 7.651)	Gasto com plano de saúde (n = 6.737)	Gasto total^a^ (n = 6.918)	Gasto com plano de saúde (n = 6.546)
Cenário econômico				
Ano de 2004	-	-	-	-
Ano de 2015^b^	0,66* (0,60 a 0,73)	0,63* (0,55 a 0,72)	0,71* (0,64 a 0,79)	0,32* (0,28 a 0,37)

IC95%: intervalo de confiança de 95%.

Nota: controlado para: sexo da criança; tipo de parto; peso ao nascer; hospitalização; criança considerada saudável; número de moradores do domicílio; mãe considerada saudável; chefe da família; idade da mãe; escolaridade da mãe; cor da pele da mãe; e tabagismo materno durante a gravidez.

^a^ Exceto o gasto com plano de saúde.

^b^ Cenário negativo, choque econômico.

* Valor p < 0,001.

Na segunda etapa da estimação, onde é considerado o valor esperado dos gastos com saúde das crianças após identificado que algum gasto de fato foi realizado, observou-se que o período de recessão econômica teve impacto significativo nestes valores. Para as crianças de 12 meses, o gasto total em saúde, excluído o gasto com plano de saúde, teve valor esperado 15% maior quando em recessão econômica, enquanto para as crianças de 24 meses, este valor foi 14% maior. No entanto, em relação ao gasto com plano de saúde, o valor esperado no cenário de recessão econômica apresentou redução de 9% para as crianças de 12 meses (Tabela 4).

**Tabela 4:** Efeito marginal (IC95%) do cenário econômico no valor esperado dos gastos com saúde das crianças nos acompanhamentos de 12 e 24 meses nas Coortes de Nascimentos de Pelotas (RS) de 2004 e 2015.

-	Acompanhamento 12 meses		Acompanhamento 24 meses	
	Gasto total^a^ (n = 3.925)	Gasto com plano de saúde (n = 2.020)	Gasto total^a^ (n = 3.314)	Gasto com plano de saúde (n = 1.744)
Cenário econômico				
Ano de 2004	-	-	-	-
Ano de 2015^b^	0,15* (0,07 a 0,22)	-0,09** (-0,17 a -0,01)	0,14** (0,06 a 0,22)	-0,01 (-0,10 a 0,08)

IC95%: intervalo de confiança de 95%.

Nota: controlado para: renda do domicílio; sexo da criança; tipo de parto; peso ao nascer; hospitalização; criança considerada saudável; mãe considerada saudável; chefe da família; idade da mãe; escolaridade da mãe; cor da pele da mãe; e tabagismo materno durante a gravidez.

^a^ Exceto o gasto com plano de saúde.

^b^ Cenário negativo, choque econômico.

* valor p < 0,001.

** valor p < 0,05.

Por fim, quanto aos controles utilizados, a renda do domicílio e o parto por cesariana indicaram relação direta com o valor esperado dos gastos em todos os cenários – aumentando o valor esperado dos gastos à medida que aumenta a renda do domicílio ou tendo sido o parto realizado por cesariana, enquanto a criança ser saudável apresentou relação inversa com gasto total, exceto plano de saúde –, diminuindo o valor esperado do gasto à medida que a criança se mostrar mais saudável.

## DISCUSSÃO

A discussão sobre os resultados encontrados demanda adaptação, uma vez que, para nosso melhor conhecimento, não há até o momento pesquisa mostrando o comportamento dos gastos diretos das famílias voltados à saúde das crianças durante uma recessão econômica – somente em relação aos gastos diretos com saúde de adultos^21^. Desta forma, usaremos estes resultados para fins de comparação, mas desde logo ressalvando sua relevante distinção.

Nosso estudo encontrou uma menor probabilidade de realização de gasto direto das famílias com a saúde das crianças durante uma recessão econômica, porém, sendo realizado algum tipo de gasto, o valor esperado deste mostrou-se superior.

O fato de que durante o período de recessão econômica há uma menor probabilidade de ser realizado algum tipo de gasto com saúde corrobora com resultados encontrados nos demais estudos realizados sobre o tema. Entretanto, nestes outros estudos, também foi menor o valor esperado do gasto durante o período de crise econômica^21^, diferentemente do encontrado em nossa análise.

Este resultado divergente, apontando uma tendência crescente em relação ao valor esperado do gasto quando realizado no período de crise econômica, pode indicar tanto uma piora do sistema público de saúde durante estes períodos, por redução do investimento público^26^, quanto uma elevação nos preços dos bens e serviços de saúde de forma geral.

Uma possível explicação para este resultado é a implementação de medidas de austeridade fiscal pelo governo no momento da recessão econômica, englobando as despesas em saúde inclusive na esfera municipal. Assim, uma vez que a crise econômica piorou as condições de saúde da população e a quantidade e qualidade dos bens e serviços de saúde disponíveis, uma resposta é o aumento do gasto direto com saúde das famílias, como também foi observado na Grécia durante a crise financeira iniciada em 2008^24^.

Importante destacar que tal relação não apresentou o mesmo comportamento em relação ao gasto com plano de saúde, uma vez que a crise econômica indicou probabilidade menor de ocorrência juntamente com menor valor esperado do gasto. Esta relação explicita o comportamento distinto do gasto com plano de saúde em relação aos demais tipos de gastos com saúde, o qual, por ser um tipo de “seguro” com pagamento mensal, permite que as famílias optem por cancelar seu pagamento face uma queda em sua renda, esperando que não aconteçam problemas de saúde que demandem sua utilização^27^.

Nesse sentido, uma menor probabilidade de ser realizado gasto com plano de saúde nas crianças no período de crise econômica, de 37% nas crianças de 12 meses e expressivos 68% nas crianças de 24 meses, corrobora este caráter preventivo dos planos^27^. Nesse ponto, mostra-se natural uma maior prevalência nos primeiros meses de vida, os quais demandam uma série de consultas médicas e exames, e uma queda brusca no avançar da idade dada a crise econômica.

O estudo apresenta limitações típicas de estudos que utilizam dados coletados referentes a gastos diretos despendidos pelos participantes. O viés de informação em relação à coleta de dados sobre gastos com plano de saúde merece maiores considerações, uma vez que o valor devidamente gasto com a criança geralmente está incluído no montante total pago em plano familiar ou sendo parcialmente subsidiado pelo empregador dos pais.

Outra importante limitação do estudo é o fato deste buscar analisar o comportamento de gastos com saúde em um contexto em que existe um sistema universal de acesso à saúde, onde diversas famílias não realizam qualquer gasto ou podem modificar livremente seu comportamento por meio de uma maior ou menor utilização desta benesse. Igualmente, devem ser consideradas potenciais diferenças nas condições sociais, econômicas e culturais entre as localidades, demandando cautela na extrapolação dos resultados para contextos mais amplos.

Cabe apontar que os programas “Brasil Sorridente”, que busca melhorar as condições de saúde bucal, e o “Farmácia Popular do Brasil”, que visa ampliar o acesso a medicamentos, foram ambos criados em 2004, sendo estes os últimos movimentos políticos com relevante impacto para a relação das famílias brasileiras com os gastos com saúde.

Desse modo, tendo tais programas impactado os dois períodos da análise e não havendo outras mudanças significativas em políticas públicas que alterassem o alcance deste sistema de saúde, em tese, podemos supor que a influência do Sistema Único de Saúde impactou igualmente ambos os períodos analisados.

Por fim, termos encontrado um resultado oposto sobre o valor esperado do gasto privado em saúde em períodos de crise econômica, comparado aos demais estudos voltados à mesma temática Ainda que justificável, ele sugere a necessidade de novos estudos para um melhor entendimento dessa relação e do impacto gerado pelo gasto público em saúde.

Essas novas pesquisas podem também estudar com mais especificidade o papel do gasto com plano de saúde para as crianças, bem como aprofundar as análises dos gastos totais em saúde, de modo a buscar interações diretas da crise econômica com seus determinantes, os quais em nossa análise foram utilizados apenas como controles.

## Data Availability

Os questionários e guias de entrevista de todas as visitas de acompanhamento estão disponíveis em [http://www.epidemio-ufpel.org.br/coorte-2004] e [http://www.epidemio-ufpel.org.br/coorte-2015].
